# The effects of fluorouracil, epirubicin, and cyclophosphamide (FEC60) on the intestinal barrier function and gut peptides in breast cancer patients: an observational study

**DOI:** 10.1186/1471-2407-13-56

**Published:** 2013-02-04

**Authors:** Francesco Russo, Michele Linsalata, Caterina Clemente, Benedetta D’Attoma, Antonella Orlando, Giovanna Campanella, Francesco Giotta, Giuseppe Riezzo

**Affiliations:** 1 Laboratory of Experimental Biochemistry, National Institute for Digestive Diseases I.R.C.C.S. “Saverio de Bellis”, Castellana Grotte, Bari, Italy; 2 Medical Oncology Unit I.R.C.C.S. “Saverio de Bellis”, Castellana Grotte, Bari, Italy; 3 Medical Oncology Unit I.R.C.C.S. “Giovanni Paolo II”, Bari, Italy; 4 Laboratory of Experimental Pathophysiology, National Institute for Digestive Diseases I.R.C.C.S. “Saverio de Bellis”, Castellana Grotte, Bari, Italy

**Keywords:** Breast cancer, Chemotherapy-induced diarrhea, Epidermal growth factor, Intestinal permeability, Ghrelin, Glucagon-like peptide 2, Gut peptides, Zonulin

## Abstract

**Background:**

Several GI peptides linked to intestinal barrier function could be involved in the modification of intestinal permeability and the onset of diarrhea during adjuvant chemotherapy. The aim of the study was to evaluate the circulating levels of zonulin, glucagon-like peptide-2 (GLP-2), epidermal growth factor (EGF) and ghrelin and their relationship with intestinal permeability and chemotherapy induced diarrhea (CTD).

**Methods:**

Sixty breast cancer patients undergoing an FEC60 regimen were enrolled, 37 patients completed the study. CTD(+) patients were discriminated by appropriate questionnaire and criteria. During chemotherapy, intestinal permeability was assessed by lactulose/mannitol urinary test on day 0 and day 14. Zonulin, GLP-2, EGF and ghrelin circulating levels were evaluated by ELISA tests at five time-points (days 0, 3, 10, 14, and 21).

**Results:**

During FEC60 administration, the lactulose/mannitol ratio was significantly higher on day 14 than at baseline. Zonulin levels were not affected by chemotherapy, whereas GLP-2 and EGF levels decreased significantly. GLP-2 levels on day 14 were significantly lower than those on day 0 and day 3, while EGF values were significantly lower on day 10 than at the baseline. In contrast, the total concentrations of ghrelin increased significantly at day 3 compared to days 0 and 21, respectively. Ten patients (27%) suffered from diarrhea. On day 14 of chemotherapy, a significant increase of the La/Ma ratio occurred in CTD(+) patients compared to CTD(−) patients. With regards to circulating gut peptides, the AUCg of GLP-2 and ghrelin were significantly lower and higher in CTD(+) patients than CTD(−) ones, respectively. Finally in CTD(+) patients a significant and inverse correlation between GLP-2 and La/Ma ratio was found on day 14.

**Conclusions:**

Breast cancer patients undergoing FEC60 showed alterations in the intestinal permeability, which was associated with modifications in the levels of GLP-2, ghrelin and EGF. In CTD(+) patients, a different GI peptide profile and increased intestinal permeability was found in comparison to CTD(−) patients. This evidence deserves further studies for investigating the potentially different intestinal luminal and microbiota conditions.

**Trial registration:**

Clinical trial NCT01382667

## Background

The complications of anti-cancer chemotherapy include gastrointestinal (GI) mucositis, which represents injury of the rest of the alimentary tract beyond oral mucositis. This condition is most prominent in the small intestine, but it also occurs in the esophagus, stomach, and large intestine [1].

GI mucositis induced by chemotherapy is associated with alterations of intestinal barrier function [2] due to the potential damage induced by the anti-cancer drugs on the epithelial cells of the intestinal mucosa. Cytotoxic drugs impair the turn-over of intestinal epithelia and induce flattening of the villi and an increased exposure of luminal contents to crypts [3]. These alterations could be involved in the frequent recurrence of GI symptoms, such as abdominal pain and diarrhea, in patients undergoing chemotherapy [4].

At present lactulose/mannitol (La/Ma) double sugar absorption test represents an appropriate, noninvasive method for evaluation of intestinal permeability. Lactulose is a disaccharide that reflects the permeability of large molecules (0.93 nm), mannitol is a monosaccharide that can be considered a marker of absorption of small molecules (0.65 nm) [5]. An increased disaccharide/monosaccharide ratio and decreased xylose absorption have been described in experimental animal models [6] and patients with GI cancer [7] treated with different cytotoxic agents [8]. Notwithstanding, there is still much to learn about the occurrence of chemotherapy-induced alterations for extra-GI neoplasms [9] and in particular with recent FEC60 regimen (fluorouracil, epirubicin, and cyclophosphamide) [10]. Furthermore, the mechanisms underlying these alterations in neoplastic patients are not fully elucidated, since there is still a lack of information concerning all of the players actively implicated in the regulation of intestinal barrier function.

Functionally, the intestinal barrier is a dynamic system, promptly responding to different stimuli ranging from the dietary state and inflammatory mediators to neuronal or humoral signals. Among the latter, zonulin is able to modulate the mucosal barrier by disassembling the tight intercellular junctions that characterize the early phase of inflammatory states [11]. This protein appears to play a key role in the pathogenesis of autoimmune diseases such as celiac disease and type-1 diabetes [12]. A tight control of the intestinal barrier is also exerted by other peptides. The Glucagon-like peptide-2 (GLP-2) is an intestinotrophic growth hormone that promotes many aspects of intestinal function, including rapid enhancements of mucosal growth [13] and the intestinal barrier function by affecting both paracellular and transcellular pathways [14]. It has been shown that GLP-2 possesses anti-apoptotic effects on intestinal crypt cells that may be useful for the attenuation of chemotherapy-induced intestinal mucositis [15]. Along with GLP-2, the epidermal growth factor (EGF) may play an active role against chemotherapy-induced damage of the intestinal mucosa. This 53 amino acid peptide shows a broad range of bioactivities on the intestinal epithelium, including the stimulation of cellular proliferation, differentiation, and intestinal maturation [16]. Previous studies have shown that EGF administration exerts a protective role against a variety of intestinal insults by either reducing injury or accelerating repair [17].

Another important molecule in regulating the mechanisms of the intestinal barrier is ghrelin, a 28 amino acid peptide mainly produced by a subset of GI enteroendocrine cells. This peptide has also been involved in the control of the mucosal barrier and is considered a potential protective agent against chemotherapy complications. In mice, ghrelin administration has been demonstrated to prevent the doxorubicin-induced GI mucosal damage [18].

The link between the modifications of circulating levels of these peptides, intestinal permeability and GI symptom profiles in extra-GI cancer patients who had undergone a second generation chemotherapy have yet to be investigated. Therefore, in the present study the La/Ma permeability test was applied to evaluate the intestinal damage in breast cancer patients when receiving the first cycle of FEC60 adjuvant chemotherapy. The effects of these cytotoxic drugs on circulating levels of zonulin, GLP-2, EGF and ghrelin were investigated. Lastly, the intestinal permeability and the gut peptide profile of patients who suffered from diarrhea during chemotherapy were also analyzed in comparison to those of patients without diarrhea.

## Methods

### Patients

This prospective observational study focused on consecutive patients with breast cancer who underwent adjuvant chemotherapy. Patients were recruited from the outpatients of the oncology D.H. of the National Institute of Digestive Diseases, I.R.C.C.S. “Saverio de Bellis” and the Medical Oncology Unit of the National Cancer Institute I.R.C.C.S. “Giovanni Paolo II”. They were screened by two investigators (G.R. and F.G.) and recruited according to the inclusion criteria and their willingness to participate. The study began in July 2010 and enrollment of patients ended in September 2011. Sixty female patients were enrolled, and 37 subjects (age 60.1±1.7 years) completed the prospective observational study.

The eligibility criteria for the study were the following: 1) a histopathologically-confirmed diagnosis of infiltrating ductal carcinoma; 2) stage II or III cancer according to the criteria of the International Union against Cancer (UICC); 3) no upper GI disease and an ability to take in soft solid foods orally; 4) Eastern Cooperative Oncology Group performance status (PS) within the range of 0 to 1.5; 5) adequate function of major organs; 6) no other active malignancy; and 7) provision of written informed consent.

Pre-treatment clinical staging was based on liver echography, computed tomography scans of the neck, chest, and the upper and lower abdomen as continuous 5 mm-thick slices and total body bone scans.

The exclusion criteria were: hypertension, diabetes mellitus and other pathologies (e.g. systemic, endocrine, collagen-related diseases). Subjects had not taken antibiotics, probiotics, vitamins, minerals, non-steroidal anti-inflammatory or prokinetic drugs, bismuth, antacids, H2-receptor antagonists, omeprazole, sucralfate or misoprostol in the 4 weeks prior to the study and had no previous history of gastric or duodenal ulcers or major GI surgery. Patients needing PPIs and/or prokinetic drugs to control their dyspeptic symptoms during the adjuvant therapy were excluded from the study at the time of analysis.

### Adjuvant chemotherapy regimen

After the surgical resection of the tumor and lymph nodes (primary therapy), all of the enrolled patients received adjuvant chemotherapy. The adjuvant regimen consisted of epirubicin-based chemotherapy. Specifically, the chemotherapy regimen consisted of FEC60 (Fluorouracil 600 mg/m2, Epirubicin 60 mg/m2 and Cyclophosphamide 600 mg/m2 every 21 days for 6 cycles).

There was no significant difference in the background of tumor status and systemic condition between patients who received adjuvant chemotherapy. Supportive therapy and prophylaxis against expected side-effects was provided. All of the patients were pre-medicated with intravenous ondansetron (4 mg), infused 1 hour prior to the administration of the scheduled drugs. Hypersensitivity reactions were prophylactically treated with intravenous dexamethasone (8 mg), which was infused 1 h prior to the administration of chemotherapy. Granulocyte-stimulating factor (G-CSF) was used for febrile neutropenia when deemed necessary.

The study was performed in accordance with the Helsinki Declaration and all participants gave written informed consent to participate before enrolment. The protocol was approved by the local Scientific and Ethics Committees and the study was registered at http://www.clinicaltrials.gov, registration number: NCT01382667.

### Blood sampling

For biochemical determinations, blood samples were collected in vacutainer tubes in the morning (at 08.00 h) after fasting from midnight and before the first cycle of chemotherapy (day 0), as well as on days 3, 10, 14 and 21 after the beginning of treatment.

### Symptom recording

During the first cycle of chemotherapy, the severity of oral mucositis was evaluated using the Oral Mucositis Assessment Scale (OMAS) on day 0 and day 14. The OMAS provides an objective assessment of oral mucositis based on the assessment of the appearance and extent of redness and ulceration in various areas of the mouth. Primary indicators were the degrees of ulceration/pseudomembrane and mucosal erythema measured in specific sites in the mouth. Secondary indicators included oral pain, swallowing, and the ability to eat as assessed by the patient [19].

Patients were evaluated for their GI symptoms with the Gastrointestinal Symptom Scoring Rate (GSRS) [20] on day 0 and day 14. The GSRS utilizes a seven-level Likert scale (1 to 7), depending on the intensity and frequency of GI symptoms experienced during the previous week. A higher score indicates more inconvenient symptoms.

During the period of observation, a list of GI symptoms was also recorded by the visual analog score (VAS) scale using a 100 mm horizontal line. Patients were asked to complete diaries in which they were to assess the intensity of a list of GI symptoms. Diaries were completed on 5 scheduled days (days 0, 3, 10, 14 and 21). Symptom scores concerned “fatigue”, “anorexia”, “nausea/vomiting”, “abdominal pain/discomfort”, “reflux disease”, and “early satiety/postprandial fullness”. In particular, with regards to “diarrhea”, patients were also asked to complete a questionnaire to assess the severity of diarrhea as well as a history of anti-diarrheal therapy at baseline and after 14 days of chemotherapy. The questionnaire incorporated eight questions to rate the severity of their diarrhea (based upon the number of bowel movements, cramping, incontinence, the impact on daily life, and duration more than one day), and also included questions to facilitate the categorization of the patient’s diarrhea according to the National Cancer Institute, Common Toxicity Criteria for Diarrhea [21].

### Intestinal permeability test

For intestinal permeability assessment, a probe solution containing 10 g lactulose and 5 g mannitol in 100 ml water was made. After an overnight fast, each subject provided a pre-test urine sample. Then they drank the test solution and collected all urine samples in the following 5 hours. Samples were stored at −20°C until analysis.

The detection and measurement of two sugar probes, La and Ma, in urine was performed by chromatographic analysis, as described by Generoso et al. [22], with a minor modification. Briefly, high-performance anion exchange chromatography coupled with pulsed amperometric detection was performed on a Dionex Model ICS-5000 with a gold working electrode and a 25 μl peek sample loop (Dionex Corp., Sunnyvale, CA, USA). Carbohydrate separation was carried out by a Carbopac PA-10 pellicular anion-exchange resin connected to a Carbopac PA-10 guard column at 30°C. Samples were eluted with 50 mM NaOH at the flow-rate of 1 ml/min. For each sample the percentage of ingested lactulose (La%) and mannitol (Ma%) in urine was evaluated and their ratio (La/Ma) was calculated. The intestinal permeability was evaluated on the day before the beginning of treatment (day 0) and on day 14 of chemotherapy administration. In a subgroup of patients (8 pts) the intestinal permeability was also evaluated on day 21.

### Zonulin, EGF, Ghrelin and GLP-2 evaluation

Serum levels of zonulin and EGF were assayed using the ELISA kits (Immunodiagnostik, Bensheim, Germany and Boster Biological Technology, Fremont, USA, respectively). The serum levels of ghrelin were assayed by the Human Ghrelin EIA kit (Ray Biotech Inc, Norcross, GA, USA).

For GLP-2 evaluation after overnight fasting, blood samples were collected in ice-chilled tubes containing 500KIU/ml of aprotinin (100,000 KIU-MP Biomedicals, LLC, Ohio, USA) and 1 mg EDTA/ml blood. The samples were centrifuged at 1600 × g for 15 min at 4°C and the separated plasma was stored at −80°C until assayed. The plasma was acidified with 1% trifluoroacetic acid (TFA, HPLC grade) and then centrifuged at 3000 × g for 20 minutes at 4°C. The supernatant was loaded onto the pre-treated C18 column (200 mg, Sep-columns, Peninsula Laboratories Inc., Belmont, California, USA). The eluent was evaporated to dryness using a centrifugal concentrator and then lyophilized. The plasma levels of GLP-2 were measured by EIA using a commercial kit (Phoenix Pharmaceuticals, Burlingame, USA).

Peptide profiles in chemotherapy patients were evaluated the day before the beginning of treatment (day 0) and at each scheduled visit on days 3, 10, 14 and 21 of the treatment.

### Statistical analysis

Data were expressed as M±SEM or median values and the range where reported. The rate of severity of diarrhea according to VAS scores was calculated to discriminate between patients reporting chemotherapy-induced diarrhea (VAS score > 0) and negative patients (VAS score = 0).

In order to evaluate the release of peptides during the time of observation, the area under the curve with respect to ground (AUCg) was calculated. The AUCg allows researchers to assess if any change occurred over time, and is indicated in the case of a profile in which data at a given point is lower than the basal value. The area under the curve was calculated using the trapezoid formula described by Pruessner et al. [23].

Parametric and non-parametric tests were applied when appropriate. The relationship between parameters was investigated by Spearman correlation analysis. Statistical significance was set at p<0.05. The software package used for the statistical analysis was StataCorp 2005 (Stata Statistical Software: Release 9, College Station, TX, USA).

## Results

### Patient characteristics

At the start of the study, the patients had a body mass index (BMI) of 27.6±5.5. When evaluated on day 0, none of the patients complained of oral mucositis (total score = 0.014±0.012) or dyspeptic symptoms (total score = 24.7±0.94) as evaluated by OMAS and GSRS, respectively. Values are expressed as M±SEM. Clinically, 40% of patients were at Stage II and 60% were at Stage III. The applied chemotherapeutic regimen for all of the patients was FEC60.

### Recurrence of oral mucositis and gastrointestinal symptoms

At day 14 of the first FEC60 cycle, none of the patients complained of oral mucositis and the OMAS score was quite similar to day 0 (day 14 = 0.017±0.061). With regards to GI symptoms, the GSRS in the whole group was higher on day 14 of chemotherapy compared to the baseline (total scores = 29.86±1.27 and 24.7±0.94, respectively, p=0.02; Paired t-test).

According to VAS and NCI Criteria, 10 patients (29%) (age = 57.6±3.99 yr.) suffered from diarrhea (Table 1), but none of them required a reduction of their chemotherapy dose. This group of patients (chemotherapy-induced diarrhea positive, CTD(+)) was considered for further evaluation in comparison to patients in the study who did not complain of this symptom (chemotherapy-induced diarrhea negative, CTD(−)). Data are expressed as M±SEM.

**Table 1:** Mean Visual Analog Scale (VAS) and National Cancer Institute Common Toxicity Criteria for Diarrhea scores and standard deviation on day 0 and day 14 of FEC60 regimen in 10 breast cancer patients who experienced diarrhea during the FEC60 cycle

**Scale**	**Day 0**	**Day 14**	**p**
VAS (0–10)	1.6 ± 0.3	3.4 ± 0.5	0.014
NCIC (0–4)	1.2 ± 0.9	1.9 ± 0.8	0.005

### Intestinal permeability tests

**Figure 1:**
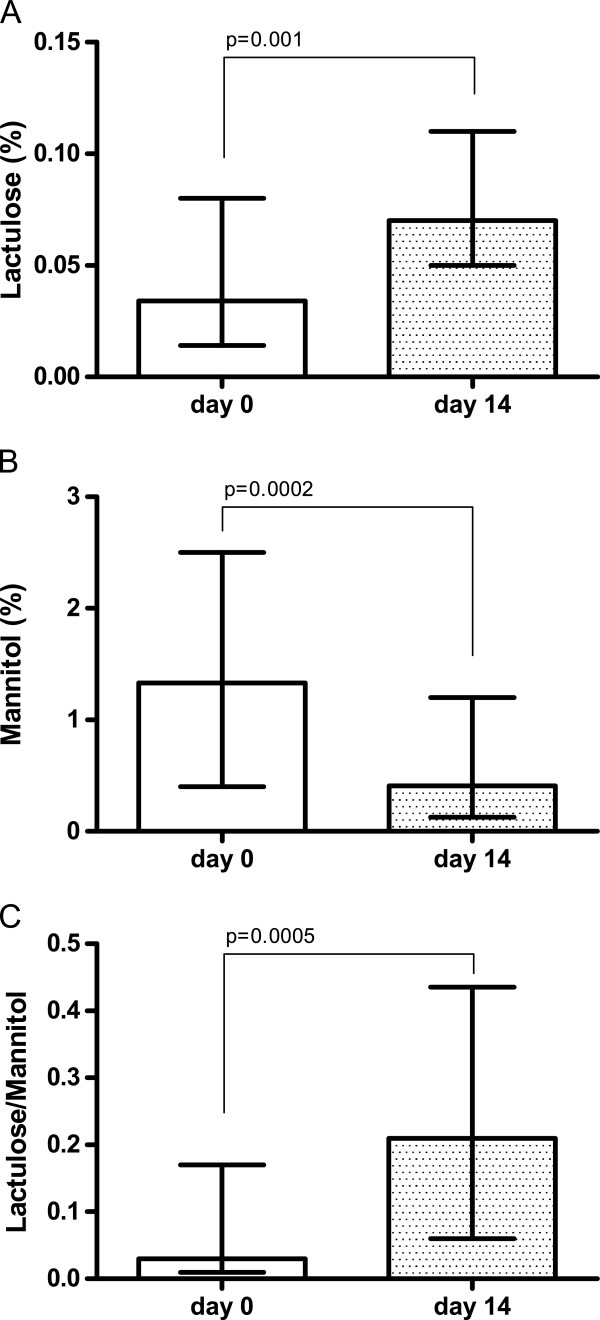
**Intestinal permeability after 14 days of FEC60.** Decreased intestinal permeability after 14 days of FEC60. Small intestinal permeability was probed by measuring the urinary cumulative 5-hour amount of lactulose (Lactulose% = percentage of ingested lactulose), mannitol (Mannitol% = percentage of ingested mannitol) and the Lactulose to Mannitol ratio. Data are reported as medians and the range and analyzed with Wilcoxon matched-pairs signed rank test.

As for small intestine permeability, La% was significantly (p = 0.001) increased 14 days after the beginning of treatment (day 14 = 0.067%; 0.005-0.82) compared to that found at baseline (day 0 = 0.034%; 0.005-0.36) (Figure 1, panel A). In contrast, Ma% was significantly (p = 0.0002) reduced after two weeks of chemotherapy (day 14: 0.41%; 0.005-4.20) compared to baseline (day 0 = 1.33%; 0.03-8.03) (Figure 1, panel B). As a result, the La/Ma ratio was significantly (p = 0.0005) increased after two weeks of chemotherapy (day 14 = 0.21; 0.06-11.43) compared to baseline (day 0 = 0.03; 0.01-2.13) (Figure 1, panel C). Data were analyzed by Wilcoxon matched pair signed rank test and expressed as median and the range.

In the group of patients (n = 8) in which the IP test was also performed on day 21 at the end of the first cycle of the FEC60 regimen, the La% and Ma% values were essentially equal to those recorded on day 14 (data not shown).

**Figure 2:**
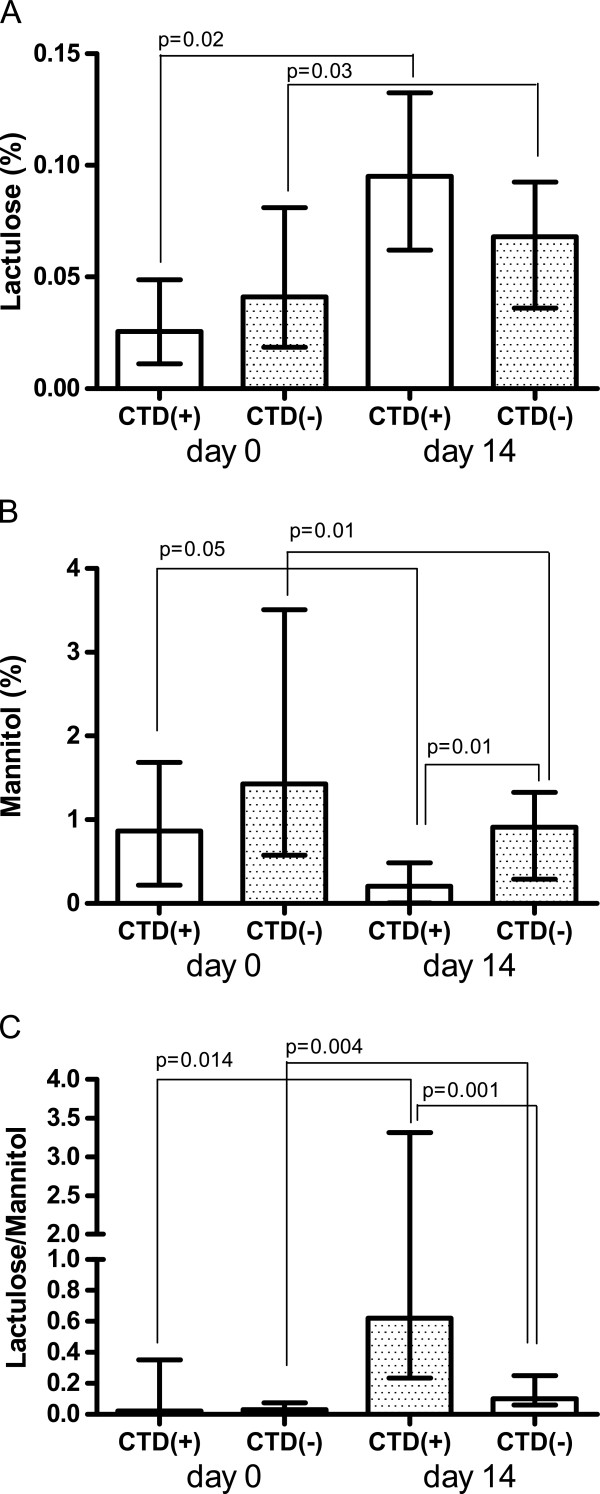
**Intestinal permeability in patients with or without chemotherapy induced diarrhea.** Intestinal permeability in patients who suffered from chemotherapy induced diarrhea, CTD(+), and those who did not, CTD(−). Small intestinal permeability was probed by measuring the urinary cumulative 5-hour amount of lactulose (Lactulose% = percentage of ingested lactulose), mannitol (Mannitol%) and the Lactulose to Mannitol ratio. Data are reported as medians and the range and analyzed with Wilcoxon matched-pairs signed rank test.

Interestingly, the intestinal barrier function, as measured by urinary recovery of the two sugars, was found to be different in CTD(+) patients from that in CTD(−) ones (Figure 2).

At day 0, La% was lower in CTD(+) patients than CTD(−) ones, even without statistical significance (0.025%; 0.005-0.21 vs. 0.041%; 0.007-0.36). After 14 days of treatment, La% increased significantly and by almost three-fold (272%) in CTD(+) patients (p = 0.02) and 65% in CTD(−) ones (p = 0.03) as well (0.093%; 0.005-0.33 vs. 0.068%; 0.007-0.82). However, at day 14 the differences between the two groups were not significant (Figure 2, panel A).

Also the Ma% recorded on day 0 was lower in CTD(+) patients than CTD(−) patients, without reaching statistical significance (0.865%; 0.033-2.30 vs. 1.43%; 0.038-8.03).

On day 14, Ma% in CTD(+) patients dramatically and significantly decreased by 76.3% in comparison to baseline values (p = 0.05), whereas in CTD(−) patients the decrease was equal to 36% (p = 0.01) (0.205%; 0.0055-0.93 vs. 0.910%; 0.007-4.20). The difference in the percentage of urinary recovery of mannitol between CTD(+) and CTD(−) patients at day 14 was statistically significant (p=0.01) (Figure 2, panel B).

With regards to the La/Ma ratio, before the start of treatment on day 0, there was no significant difference between the two groups (0.02; 0.01-0.75 vs. 0.03; 0.01-2.13). After two weeks of treatment, the La/Ma ratio increased dramatically and significantly by several-fold in both CTD(+) patients (p = 0.014) and CTD(−) ones (p = 0.004). Importantly, the La/Ma ratio of CTD(+) patients was significantly higher (p = 0.001) than that recorded in CTD(−) group (0.62; 0.11-11.43 vs. 0.10; 0.02-1.6) (Figure 2, panel C).

Data were analyzed by the Wilcoxon rank sum test for differences within the groups and the Mann Whitney test for differences between the groups, and results were expressed as median and the range.

### Gastrointestinal peptides

**Figure 3:**
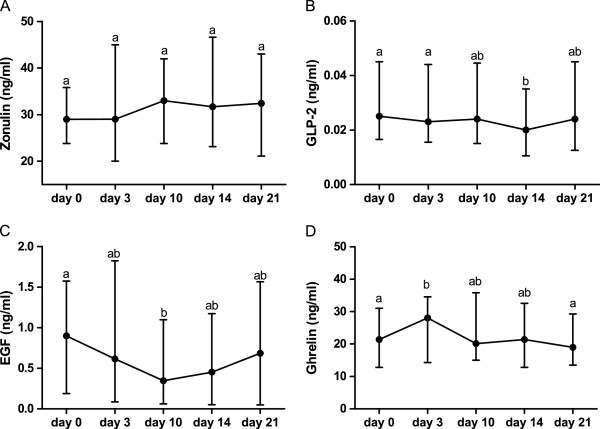
**Basal plasma values of gut hormones during FEC60 protocol.** Basal plasma values of gut hormones at baseline (day 0) and during the scheduled days (day 3, day 10, day 14, day 21). Values not showing a common letter differ significantly. Data are reported as median and the range and analyzed with Friedman test and Dunn’s Multiple Comparison Test.

Figure 3 panel A shows the serial changes in total zonulin concentration. The median serum total zonulin levels did not change significantly compared to baseline during the FEC60 cycle (day 0 = 28.9; 4.61-100.9 ng/ml; day 3 = 29.0; 0.89-100 ng/ml, day 10 = 33.0; 2.0-102 ng/ml; day 14 = 31.70; 6.7-104 ng/ml; day 21 = 32.44; 0.74-104 ng/ml).

The circulating levels of GLP-2 (Figure 3, panel B) decreased significantly during the chemotherapy regimen (p = 0.0044). GLP-2 levels on day 14 (0.02; 0.001-0.45 ng/ml) were significantly lower (p<0.05) than those recorded on day 0 (0.025; 0.003-0.704 ng/ml) and day 3 (0.025; 0.003-0.73 ng/ml), but not than levels recorded on day 10 (0.024; 0.003-0.45 ng/ml) and day 21 (0.024; 0.003-0.80 ng/ml).

The EGF concentrations are shown in Figure 3, panel C. A significant decrease during the FEC60 cycle occurred (p = 0.004). EGF values on day 10 (0.347; 0.03-2.94 ng/ml) were significantly lower (p<0.05) than those recorded on day 0 (0.90; 0.03-3.88 ng/ml), but not than levels recorded on day 3 (0.615; 0.03-3.17 ng/ml), day 14 (0.45; 0.025-3.45 ng/ml), and day 21 (0.69; 0.025-3.61 ng/ml).

With regards to ghrelin (Figure 3, panel D), the total concentration at day 3 (28.06; 14.26-53.0 ng/ml) increased significantly (p<0.05) by 14.6% and 17.6% compared to levels recorded at day 0 (21.38; 12.83-49.12 ng/ml) and after completion of chemotherapy on day 21 (18.97; 13.48-58.34 ng/ml), respectively. In contrast, there were no significant differences between day 3 and day 10 (20.16; 15.01-60.89 ng/ml) and day 14 (21.38; 12.83-58.34 ng/ml). Data are expressed as median and the range and analyzed with Friedman test and Dunn’s Multiple Comparison Test. No correlation between intestinal permeability and GI peptides was found.

**Figure 4:**
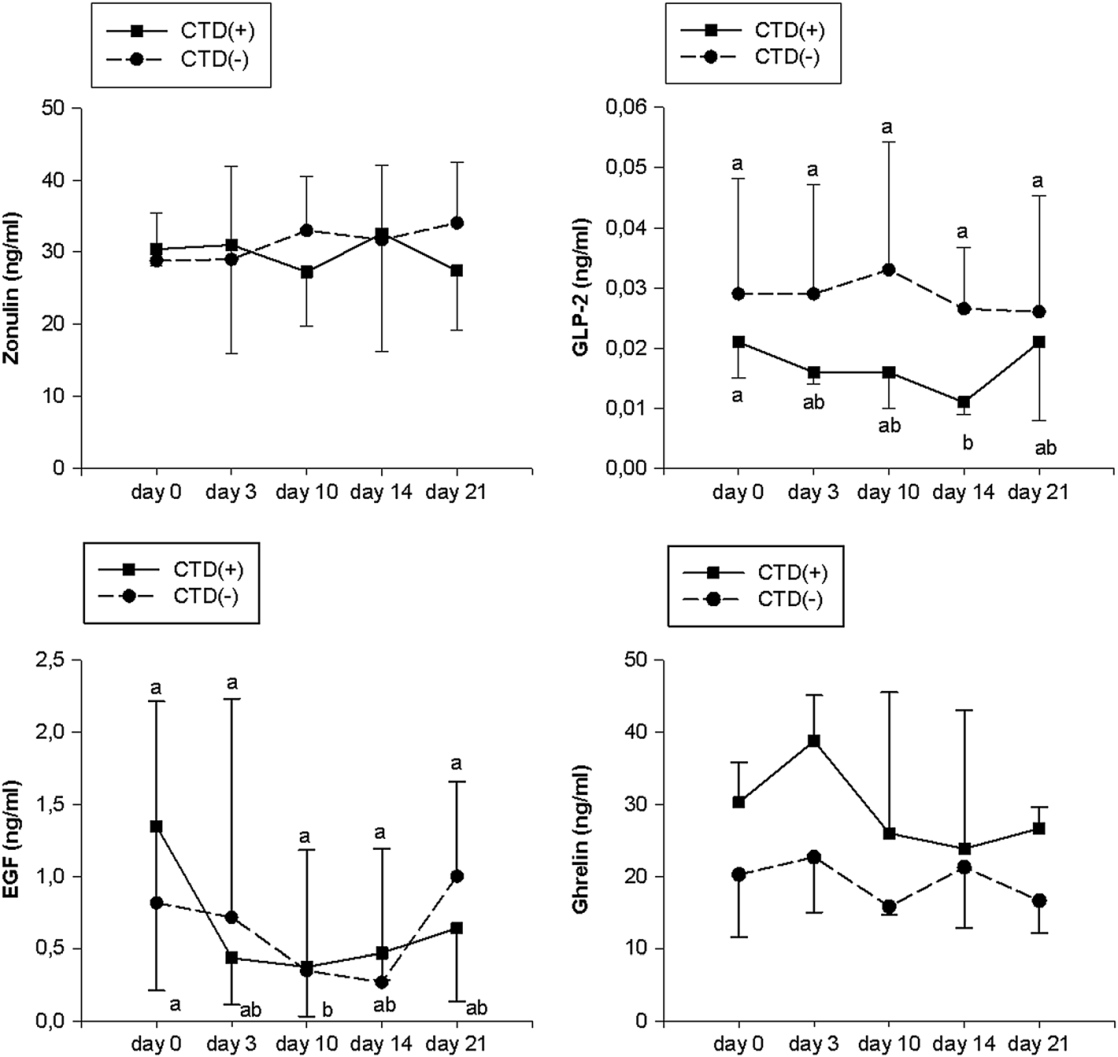
**Gut hormone profiles in patients with or without chemotherapy induced diarrhea.** Basal plasma values of gut hormones at baseline (day 0) and during the scheduled days (day 3, day 10, day 14, day 21) in patients who suffered from chemotherapy induced diarrhea, CTD(+), and those who did not, CTD(−). Values not showing a common letter differ significantly. Data are reported as median and the range and analyzed with Friedman test and Dunn’s Multiple Comparison Test.

The gut peptide profile of CTD(+) patients was also analyzed in comparison to that of CTD(−) ones. Zonulin and ghrelin levels did not change significantly compared to starting values in both groups (Figure 4, panel A and D). With regards to GLP-2 and EGF circulating concentrations, the gut peptide profile of CTD(+) patients was found to be different from that of CTD(−) ones (Figure 4, panel B and C). In CTD(+), but not in CTD(−) patients, GLP-2 levels on day 14 (0.011; 0.001-0.050 ng/ml) were significantly lower (p<0.05) than those recorded on day 0 (0.021; 0.008-0.07 ng/ml). Similarly, EGF values on day 10 (0.373; 0.03-1.13 ng/ml) were significantly lower (p<0.05) than those recorded on day 0 (1.348; 0.098-2.313 ng/ml). Data are expressed as the median and range and were analyzed with Friedman test and Dunn’s Multiple Comparison Test.

In order to calculate the differences of the total release of these peptides during the FEC60 cycle, the AUCg values of zonulin, GLP-2, ghrelin and EGF were calculated. The AUCg of zonulin were similar in the two groups (621.7; 225.5-2148 vs. 662; 105.9-1647) whereas the GLP-2 levels were significantly (p = 0.04) lower in the CTD(+) group than in the CTD(−) one (0.33; 0.09-0.85 vs. 0.66; 0.13-12.45). The AUCg values of EGF were not significantly different in the two groups (16.59; 1.33-25.69 vs. 15.57; 0.74-68.65). By contrast, the AUCg values of ghrelin were significantly higher (p = 0.0427) in the CTD(+) group compared to those in the CTD(−) one (588.3; 275.8-1098 vs. 410.3; 152.8-985.4). Data were analyzed by the Mann Whitney test and expressed as median and the range.

**Figure 5:**
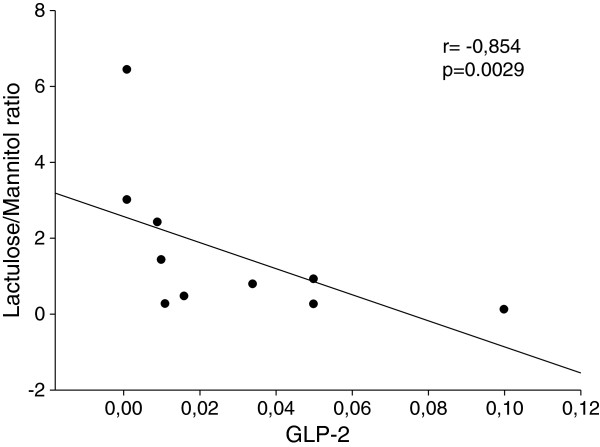
**Correlation between Lactulose to Mannitol ratio and GLP-2.** Negative and significant correlation (r = −0.854, p = 0.0029 Spearman correlation coefficient) between intestinal permeability as expressed by Lactulose to Mannitol ratio and the circulating levels of GLP-2 recorded on day 14 in the group of patients who suffered from chemotherapy induced diarrhea, CTD(+).

Finally, in the CTD(+) group a negative and significant correlation between the La/Ma ratio and the circulating levels of GLP-2 recorded on day 14 was found (r = −0.854, p = 0.0029, Spearman correlation coefficient) (Figure 5).

## Discussion

Cytotoxic drugs may induce mucosal modifications leading to an increased contact between luminal fluid and the cryptal epithelium. Our study reports a significant change of intestinal permeability mirrored by a significant raise of the La/Ma ratio recorded on day 14, and persisting until the end of the first cycle of chemotherapy. It appeared to be determined by a concomitant increase in La% and decrease in Ma%. In this condition, the passive transport of monosaccharides decreases, while more disaccharides may cross the mucosa through large pores [8]. This finding is in line with data showing an involvement of cytotoxic drugs in the processes regulating macromolecular passage through both paracellular and transcellular pathways.

To the best of our knowledge, this is the first attempt to evaluate the existence of effects due to the FEC60 regimen on the intestinal barrier functioning and the release of peptides involved in the regulation of intestinal permeability in cancer patients. FEC60, based on 5FU, cyclophosphamide, and epirubicin is now the most commonly used FEC regimen as adjuvant treatment for breast cancer patients. It is active and well tolerated but, apart from leukopenia, common adverse events include the increase in liver enzymes and a series of GI symptoms, such as nausea, vomiting and diarrhea [24]. In this framework, the breast cancer patients involved in this study had a relatively fair systemic status and stable tumor progression. Furthermore, they did not suffer from a direct involvement of the GI tract, and so they represented an appropriate cohort for investigating the above issues.

Data in the literature suggest that, where permeability is increased, a common pathophysiological event is the up-regulation of zonulin secretion from a lamina propria source into the lumen with an inappropriate activation of this pathway. As a consequence, the end result is increased paracellular permeability [25]. However, in the patients studied here, zonulin levels were not affected by chemotherapy, which is a finding that is not completely surprising since previous studies performed on laboratory animals have already demonstrated that chemotherapy is able to increase IP without affecting the expression or the circulating levels of zonulin [26].

Available reports strongly support a key role for endogenous peptides in promoting an adequate trophic status in intestinal mucosa to help maintain its barrier function [27]. In this connection, different GI peptides have been considered therapeutic tools in gut diseases and some of them, especially those that have a trophic action on the intestinal mucosa, like GLP-2 and EGF, have been identified for their potential clinical use [28]. However, there is still little information about circulating levels of these peptides during chemotherapy in humans. Present data show that the circulating levels of GLP-2 decreased significantly during chemotherapy, reaching the lowest level on day 14 in comparison to the baseline. This happened in conjunction with the significant increase of intestinal permeability. This datum is in accordance with studies showing that GLP-2 enhances intestinal epithelial barrier function by affecting both paracellular and transcellular pathways in mice [29]. Also, EGF was found to decrease significantly during chemotherapy, reaching the lowest circulating concentration at day 10. This lowering corresponds with the reported peak in the occurrence of mucositis [8]. EGF is considered to be an effective mitogen for intestinal mucosa [16]. Additionally, treatment with EGF before and after irradiation has been proven to limit the manifestations of mucositis [16]. Our data for GLP-2 and EGF seem in agreement with the assumption of a combined action of these two peptides. As a matter of fact, the biology of GLP-2 in the gut overlaps to a considerable extent with the actions of ErbB ligands (including EGF, along with other peptides such as betacellulin, TGF-alpha, amphiregulin) as well as their receptors [30]. Both EGF and GLP-2 stimulate cell proliferation [31,32], enhance mucosal adaptation following small bowel resection [16,33] and accelerate recovery of epithelial integrity following radiation-induced injury [34,35]. Besides, concomitant administration of GLP-2 and EGF has additive effects increasing small intestinal weight, cellular proliferation, and morphometric measurements of villus and crypt area [36].

A differing behavior was observed for ghrelin that rapidly rouse on day 3 of treatment and rapidly lowered to normal values on day 10. This peptide represents the natural endogenous ligand for GH secretagogue receptors (GHS) originally isolated from rat and human stomach [37], and subsequently identified in various tissues, including the small bowel [38]. Diseases of the intestines, including inflammatory processes, can modify ghrelin levels [39,40]. It has been suggested that the inhibition of ghrelin secretion during inflammatory processes might potentiate the ongoing inflammatory insult [39]. The rapid increase in ghrelin levels observed on day 3 may be concomitant with the initial phase of the mucositis process in the gut [1], thus representing an acute response against the damage induced by cytotoxic drugs. Overall, these data led to the hypothesis of a strong implication for GI peptides in the occurrence of intestinal barrier dysfunction during the administration of chemotherapeutic regimens to breast cancer patients.

Of note, the changes in circulating gut peptides and the alterations of intestinal permeability appeared to be associated with clinical manifestations of GI toxicity, the global symptom profile being affected by the first chemotherapy cycle, and 10 out 37 patients (27%) suffered from diarrhea. Diarrhea is one of the most common chemotherapy side-effects [41] and different mechanisms may be involved in its pathogenesis reflecting an imbalance between the secretory activity of cryptal epithelial cells and absorption by the villar epithelium [8].

Firstly, a dramatic and significant increase of intestinal permeability in CTD(+) patients but not in CTD(−) ones occurred on day 14. Secondly, CTD(+) patients exhibited a lower GLP-2 profile compared to CTD(−) ones, reaching the lowest level on day 14. On the contrary, ghrelin levels were higher in CTD(+) patients than CTD(−) ones, reaching the peak on day 3. Lastly, a significant and negative correlation between the lactulose to mannitol ratio and GLP-2 levels recorded on day 14 was found in CTD(+) patients. Taken as a whole, these results agree with other data in the literature showing an involvement of GLP-2 and ghrelin in the onset of diarrhea following alterations of the intestinal barrier function.

It has been reported that mice with a disruption of the prohormone convertase-1 gene exhibited marked reductions in the intestinal levels of GLP-2, reduced somatic growth, and diarrhea, suggesting that GLP-2 action may contribute to regulation of murine intestinal function in vivo [42]. Besides, a therapeutic role for GLP-2 in the management of diarrhea induced by cytotoxic drugs has been hypothesized. Physiologically, GLP-2 delays gastric emptying, perhaps by inhibiting centrally induced antral motility [43], thereby acting as one of the mediators of the so-called ileal brake. The global combination of intestinetrophic effects, functional improvement, and an enterogastrone effect have made GLP-2 promising as an agent also for the treatment of cancer patients suffering from diarrhea, and the findings of this study seem to further support this novel therapeutic indication [44]. As far as ghrelin is concerned, previous papers have demonstrated that patients with GI diseases affecting intestinal barrier functions, such as celiac disease, showed high hormone levels compared to healthy controls. In addition, ghrelin levels correlated significantly with the degree of the severity of intestinal mucosal lesions. Present findings substantiate the hypothesis that there is an inter-play of hormonal, metabolic, and nutritional factors that could also influence ghrelin secretion under pathophysiological circumstances [42].

## Conclusions

It could be supposed that in patients with extra GI neoplasms, such as breast cancer, the FEC60 protocol induces alterations in the intestinal permeability associated with modifications in the levels of those hormones regulating the growth and physiology of intestinal membranes (such as GLP-2, ghrelin and, to a less extent, EGF). In the group of patients with diarrhea, a different GI peptide profile and increased intestinal permeability were found.

Further studies will be performed to confirm the present data and to investigate the potentially different susceptibility of patients undergoing chemotherapy who have diarrhea due to possible intestinal luminal conditions (e.g. peculiar microbiota and/or possible shifts in the intestinal microbial populations).

## Abbreviations

GI: Gastrointestinal; FEC60: Fluorouracil, Epirubicin, Cyclophosphamide; GLP-2: Glucagon-like peptide-2; EGF: Epidermal Growth Factor; UICC: International Union against Cancer; G-CSF: Granulocyte-stimulating factor; OMAS: Oral Mucositis Assessment Scale; GSRS: Gastrointestinal Symptom Scoring Rate; VAS: Visual analog score; NCI: National Cancer Institute; La%: Percentage of ingested lactulose in urine; Ma%: Percentage of ingested mannitol in urine; La/Ma: Lactulose to Mannitol ratio; ELISA: Enzyme-Linked ImmunoSorbent Assay; TFA: Trifluoroacetic Acid; EIA: Enzyme Immunoassay; AUCg: Area under the Curve with respect to ground; BMI: Body Mass Index; CTD: Chemotherapy Induced Diarrhea; GHS: GH Secretagogue receptors.

## Competing interests

The authors declare that they have no competing interests.

## Authors’ contributions

F.R. and M.L. contributed equally to this work. G.R., F.R. and M.L. designed the research. G.R., F.G. and G.C. enrolled patients. C.C., A.O. and B.D. analyzed the data. G.R., F. R. and M.L. designed the research and wrote the paper. All authors read and approved the final manuscript.

## Pre-publication history

The pre-publication history for this paper can be accessed here:

http://www.biomedcentral.com/1471-2407/13/56/prepub
